# Evidence for Association of the E23K Variant of *KCNJ11* Gene with Type 2 Diabetes in Tunisian Population: Population-Based Study and Meta-Analysis

**DOI:** 10.1155/2014/265274

**Published:** 2014-07-07

**Authors:** Khaled Lasram, Nizar Ben Halim, Sana Hsouna, Rym Kefi, Imen Arfa, Welid Ghazouani, Henda Jamoussi, Houda Benrahma, Najla Kharrat, Ahmed Rebai, Slim Ben Ammar, Sonia Bahri, Abdelhamid Barakat, Abdelmajid Abid, Sonia Abdelhak

**Affiliations:** ^1^Pasteur Institute of Tunis, Laboratory of Biomedical Genomics and Oncogenetics, LR11IPT05, BP 74, 13 Place Pasteur, Le Belvédère, 1002 Tunis, Tunisia; ^2^Université Tunis El Manar, 1068 Tunis, Tunisia; ^3^National Institute of Nutrition and Food Technology, Department of External Consultation, 11 rue Jebel Lakhdar, Bab Saadoun, 1007 Tunis, Tunisia; ^4^Pasteur Institute of Morocco, Department of Scientific Research, Laboratory of Molecular and Human Genetics, 1 Place Louis Pasteur, 20360 Casablanca, Morocco; ^5^Centre of Biotechnology of Sfax, Unit of Bioinformatics and Biostatistics, 3038 Sfax, Tunisia; ^6^Pasteur Institute of Tunis, Central Laboratory of Medical Biology, 13 Place Pasteur, Le Belvédère, 1002 Tunis, Tunisia

## Abstract

*Aims*. Genetic association studies have reported the E23K variant of *KCNJ11* gene to be associated with Type 2 diabetes. In Arab populations, only four studies have investigated the role of this variant. We aimed to replicate and validate the association between the E23K variant and Type 2 diabetes in Tunisian and Arab populations. *Methods*. We have performed a case-control association study including 250 Tunisian patients with Type 2 diabetes and 267 controls. Allelic association has also been evaluated by 2 meta-analyses including all population-based studies among Tunisians and Arabs (2 and 5 populations, resp.). *Results*. A significant association between the E23K variant and Type 2 diabetes was found (OR = 1.6, 95% CI = 1.14–2.27, and *P* = 0.007). Furthermore, our meta-analysis has confirmed the significant role of the E23K variant in susceptibility of Type 2 diabetes in Tunisian and Arab populations (OR = 1.29, 95% CI = 1.15–1.46, and *P* < 10^−3^ and OR = 1.33, 95% CI = 1.13–1.56, and *P* = 0.001, resp.). *Conclusion*. Both case-control and meta-analyses results revealed the significant association between the E23K variant of *KCNJ11* and Type 2 diabetes among Tunisians and Arabs.

## 1. Introduction

Type 2 diabetes is a polygenic disorder characterized by defects in insulin secretion and peripheral insulin resistance [1]. Tunisia, as many countries worldwide, is increasingly affected by diabetes. In the Tunisian population, the prevalence of Type 2 diabetes mellitus (T2DM) reaches 9% among adults [2] against 2.3% in 1977 [3]. These values showed an intermediate level compared to several human populations but they are still higher compared to European populations. The increased level of T2DM may be due to the rapid and recent lifestyle changing and/or a specific genetic background.

It is well established that the potassium inwardly rectifying channel, subfamily J, member 11 (*KCNJ11*) gene located at chromosome 11p15.1 is involved in insulin secretion in humans and that mutations in this* KCNJ11* gene can cause congenital hyperinsulinism [4] and permanent neonatal diabetes [5]. Consequently,* KCNJ11* has been so far investigated as T2DM candidate gene.

In 2003, the E23K variant was shown to be a robustly associating T2DM susceptibility variant [6] and, in 2005, the relationship between this variant and T2DM has been fully elucidated. In fact,* KCNJ11* gene encodes a pore-forming subunit of the inwardly rectifying ATP-sensitive K+ channel (Kir6.2), which is one component of the ATP-sensitive potassium (K_ATP_) channels in pancreatic *β*-cells. K_ATP_ channels regulate insulin secretion by coupling the metabolic state of the cell to membrane potential. Elevation of blood glucose level leads to an increase in the ATP to ADP ratio and a decrease in K_ATP_ channel permeability that in turn leads to membrane depolarization, activation of voltage-dependent calcium channels, Ca^2+^ influx into the cell, and finally insulin exocytosis [7].

However, the reported functional studies are not yet categorical and the full mutational mechanism(s) is still incomplete [8] due to the difficulty in identifying the causal variant. This is particularly the case when a second nonsynonymous variant in the neighboring* ABCC8* gene (rs757110) was found in complete linkage disequilibrium with the E23K variant [9].

The E23K variant has been reported to be associated with T2DM in various ethnic populations, including European-descent populations [6, 9, 10] and Asians [11–13]. Among Arab populations, the results were controversial [19–22]. However, these findings cannot be extrapolated to other populations particularly in the Tunisian population which is made of a mosaic of communities and described as genetically heterogeneous.

Thus, we proposed in this study to investigate the role of the E23K variant of* KCNJ11* gene in the development of T2DM in the Tunisian population.

## 2. Methods

Details of genotyping data for each subject are given in Table 1. Written informed consent was obtained from all subjects.

### 2.1. Study Population

The study population consisted of 250 unrelated Tunisian patients with T2DM (62.4% females; average age at recruitment 60.2 ± 10.5 years; average age at T2DM diagnosis 45.6 ± 9.5 years) and 267 control subjects (60.7% females; average age at recruitment 53.7 ± 11.6 years) (Table 1).

Subjects with T2DM were diagnosed according to the World Health Organization (WHO) criteria [14] and recruited from National Institute of Nutrition and Food Technology (INNTA), the referral diabetes Medical Center in Tunisia. Subjects with positive glutamic acid decarboxylase antibodies were excluded.

Population control subjects were chosen (1) older than 40 years, age described as significantly related to diabetes in Tunisian population [2], (2) without first degree family history of diabetes given the high familial aggregation within this category compared to the second degree relatives [15], and (3) with normal (<6.1 mmol/L) fasting plasma glucose.

This study is a part of an international cooperative project, NEPAD/NABNet_T2D_NA (New Partnership for Africa's Development, North Africa Biosciences Network, Type 2 diabetes, North Africa), which aims to assess the involvement of the E23K variant of* KCNJ11* with T2DM in four North African populations (Tunisia, Algeria, Mauritania, and Morocco). In the present work, we have evaluated the association of the E23K variant in 250 T2DM patients and 250 controls from Tunisian population.

### 2.2. Genetic Analyses

#### 2.2.1. Blood Sample Collection and DNA Extraction

Peripheral blood was drawn from T2DM patients and controls in ethylenediaminetetraacetic acid (EDTA) anticoagulated tubes. DNA was extracted from the blood using salting-out protocol [16].

### 2.3. Genotyping

Genotyping of E23K variant was carried out using a TaqMan allelic discrimination assay. The assay was carried out using an ABI Prism 7500 Sequence Detection System (Applied Biosystems, Foster City, CA, USA) in a reaction volume of 20 *μ*L, according to the manufacturer's instructions and using commercially available primers and probes. A random sample set of 10% was retested with the same method to confirm genotype accuracy. The genotyping success rate was 97.13% for cases and controls and the concordance rates were 100% in duplicate samples.

### 2.4. Statistical Analysis

Hardy-Weinberg equilibrium (HWE) analyses were performed on *R* using SNP-HWE program [17]. Comparisons between cases and controls for quantitative traits were performed using Student's *t*-test. For qualitative traits we used chi-square test. We tested E23K variant allelic and genotypic association with T2DM risk using multivariate logistic regression to adjust for age, gender, and body mass index (BMI). Odd ratios (ORs) with 95% confidence interval (95% CI) were assessed for the risk allele. For the genotyping data we tested various genetic models including additive, dominant, and recessive models. Allelic exact test was computed on *R* using allelic package. Analysis of variance (ANOVA) or the unpaired two-tailed Student's *t*-test was performed to compare clinical data. Multivariate linear regression was used to test the association of the E23K variant with quantitative traits. A *t*-stat of greater than 1.96 with significance less than 0.05 indicates that the independent variable is a significant predictor of the dependent variable. Statistical analyses were performed using Stata 11 software (StataCorp, College Station, TX, USA). A *P* value < 0.05 was considered statistically significant. Power calculations were performed using PS (Power and Sample Size Calculations software (version 3.0)) [18]. Based on probability of exposure in controls obtained in our study (0.19), we had 56.9% power to detect ORs of 1.68 at *P* < 0.05.

### 2.5. Meta-Analysis

We further collected data from the literature for E23K variant in the* KCNJ11* gene in Arab populations by searching PubMed using key words:* KCNJ11*, Arab, E23K, and Diabetes. Four studies were identified after literature search. Three reports were combined to our study data as the last report by Mtiraoui et al. [20] was a replication of the previous study reported by Ezzidi et al. [19] by increasing the sample size. As the study by Mtiraoui did not report genotyping data, we performed an allelic meta-analysis. Statistical heterogeneity between studies was tested by chi-square-based *Q* test and quantified using *I*-squared test. The fixed-effects method of Mantel-Haenszel was used when *P*_heterogeneity_ > 0.10 or *I* ^2^ < 50%. Otherwise, a random-effects model of DerSimonian and Laird was used. The conservative Egger's regression analysis was used to evaluate publication bias between the considered 3 studies. Begg's test was used when just two studies are pooled. All statistical analyses were conducted by using STATA software version 11.0.

## 3. Results

### 3.1. Case-Control Study

Genotypic distribution of E23K did not show any deviation from Hardy-Weinberg equilibrium, *P* = 0.32 in T2DM patients and *P* = 0.69 in controls (Table 2). The observed clinical and biochemical data are shown in Table 1. The mean age of the diabetic group (60.2 ± 10.5) was higher (*P* < 10^−4^) than that observed among controls (53.7 ± 11.76). Systolic blood pressure, fasting glucose, and triglycerides means were significantly higher in T2DM patients than in controls (*P* < 10^−4^). The high density lipoprotein-cholesterol (HDL-C) distribution was significantly higher in controls than in T2DM patients (*P* < 10^−4^). Sex ratio did not significantly differ between the two groups (*P* = 0.65).

The frequency of the K allele is significantly higher in cases than in controls (25.4% versus 19%; *P* = 0.015). The K allele is significantly associated with susceptibility to T2DM (OR = 1.44, 95% CI = 1.07–1.93, and *P* = 0.017) (Table 2).

Logistic regression analysis adjusted for age, gender, and BMI shows a significant association between the E23K variant and T2DM (OR = 1.6, 95% CI = 1.14–2.27, and *P* = 0.007) (Table 2). The E23K variant increased the risk of T2DM in the additive model (EE versus KK, OR = 3.68, 95% CI = 1.28–10.58, and *P* = 0.016), in the dominant model (OR = 1.61, 95% CI = 1.07–2.42, and *P* = 0.022), and in the recessive model (OR = 3.23, 95% CI = 1.13–9.21, and *P* = 0.028) (Table 2).

### 3.2. Association of E23K Variant with Quantitative Traits

When examining the association between the E23K variant and various T2DM-related quantitative traits (fasting plasma glucose, BMI, total cholesterol, triglycerides, HDL-C, systolic blood pressure, diastolic blood pressure, and low density lipoprotein-cholesterol (LDL-C)) among T2DM group, control group, and the overall sample, fasting plasma glucose values were found to differ significantly across the E23K genotypes in T2DM and in the pooled sample, *P* = 0.04 and *P* = 0.03, respectively (Table 3). Fasting plasma glucose levels were higher in subjects with the EK genotype (12.49 ± 4.13) than in those with the KK (10.81 ± 4.48) and EE genotypes (11.03 ± 3.89) in T2DM group. When considering the overall sample, a different tendency was observed and fasting plasma glucose levels were higher in subjects with the KK genotype (9.54 ± 4.53) than in those with the EK (9.32 ± 4.81) and EE genotypes (7.93 ± 3.93). However, no significant differences were observed between genotypes and groups for BMI, total cholesterol, triglycerides, HDL-C, systolic blood pressure, and diastolic blood pressure.

Multiple regression analyses were performed to assess the independent contribution of the statute (presence or absence of T2DM), the age, the gender, and the BMI to fasting plasma glucose. E23K genotype of the* KCNJ11* gene was found to be independently related to fasting plasma glucose levels under a dominant model: fasting plasma glucose explained 65% (*P* = 0.031) of the E23K variability of E23K (Table 4).

### 3.3. Tunisian Meta-Analysis

**Figure 1:**
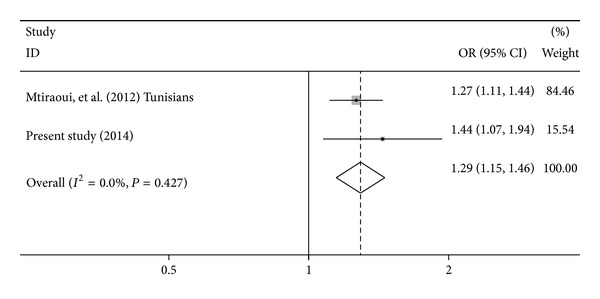
Meta-analysis of the association between* KCNJ11* E23K and T2DM in Tunisian population. Forest plot of allelic odds ratio (OR). The area of the squares reflects the study specific weight. The diamond shows the summary fixed-effects odds ratio estimate from 2 studies.

We first pooled overall effects of the E23K variant and T2DM risk of 1720 Tunisian patients and 1105 control subjects from the current study with the one from Central East Tunisia of Mtiraoui et al. [20]. The pooled OR for allelic frequency comparison suggested that the E23K variant is significantly associated with an increased T2DM risk: OR = 1.29, 95% CI = 1.15–1.46, *P* < 10^−3^, *P*_heterogeneity_ = 0.427, and *I* ^2^ = 0.0% (fixed effects) (Figure 1). Begg's test showed that there is no publication bias for E23K variant (*P* = 0.317).

### 3.4. Arab Meta-Analysis

We extended the analysis to Arab populations and performed a meta-analysis of the E23K variant on 3156 T2DM patients and 2493 controls. For this purpose, we have added results from a Saudi study realized in 2007 [21], the Lebanese participants of the Mtiraoui et al. study [20], and a recent Mauritanian study [22] to the two previous Tunisian samples.

**Figure 2:**
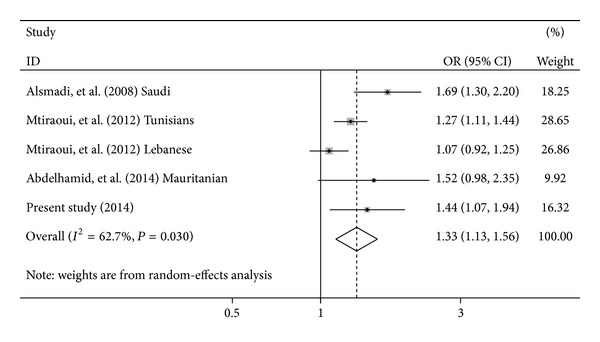
Meta-analysis of the association between* KCNJ11* E23K and T2DM in Arab population. Forest plot of allelic odds ratio (OR) under the random-effects model. The area of the squares reflects the study specific weight. The diamond shows the summary odds ratio estimate from 5 studies.

The pooled OR for allelic frequency comparison suggested that the E23K variant is significantly associated with an increased T2DM risk: OR = 1.33, 95% CI = 1.13–1.56, *P* = 0.001, *P*_heterogeneity_ = 0.03, and *I* ^2^ = 62.7% (random effects) (Figure 2). Egger's test showed that there is no publication bias (Egger's test) for E23K variant (*P* = 0.228).

## 4. Discussion

In the present study, we sought to replicate the association of the E23K variant in* KCNJ11* gene identified in the aforementioned studies [6, 9, 10, 20–22] with T2DM in an independent Tunisian case-control sample. We validated the positive association between E23K and T2DM in the Tunisian population with an increased risk of 1.6. Interestingly, T2DM subjects with the EK genotype had more severe hyperglycemia than subjects with EE or KK. Likewise, controls carrying the KK genotype had the highest fasting plasma glucose level, suggesting genotype-phenotype correlation as reported by Schwanstecher et al. [23]. In the former study, the heterozygous (EK) and homozygous (KK) state of E23K variant promote the development of T2DM by increasing the ATP concentration threshold that induces both pancreatic *β*-cells K_ATP_ channels overactivity and insulin secretion inhibition [23]. Nielsen et al. [10] have also reported the association of E23K variant with decreased levels of insulin secretion in nondiabetic individuals.

We also found that the KK genotype confers a 3.62 times higher risk for T2DM compared to the EE genotype. Our findings were in contrast with several studies on Caucasians [6, 9, 10] and Chinese Han [13] showing a significant association between the K23 allele and T2DM under a recessive model. Our results were also not in accordance with some Asian studies on Japanese [11] and Korean populations [12], where a strong association has been reported between the K23 allele and T2DM under a dominant model. Few studies were performed on the involvement of the E23K variant on T2DM pathogenesis in Arab populations. Alsmadi et al. [21] have shown a significant association of the K23 allele under an additive model (EE versus EK) while it has been reported under a dominant model in the Mauritanian population [22]. These controversial results show specific ethnic differentiation of the E23K variant effect under different genetic models. In contrast to results found in Mtiraoui et al. [20], study on population from Central East Tunisia revealed no significant association [19]. This discrepancy might be explained by the population stratification in the Central East of Tunisia. Indeed, a replication study with a larger sample size showed a positive association [20]. The former study reported no significant association in the Lebanese population [20]. In our studied sample, the K minor allele frequency (MAF) of the E23K variant was 0.25 in cases and 0.19 in controls (Table 2), while in Mtiraoui et al., it was found at 0.33 in cases and 0.28 in controls [20]. Similarly, in Alsmadi et al., the MAF of E23K variant was 0.21 in cases and 0.14 in controls [21]; in Abdelhamid et al., it was of 0.22 in cases and 0.16 in controls [22]. In Caucasian populations, such as the Danish and British, the MAF of the E23K variant seems to be more homogenous among cases and controls with a frequency of 0.4 and 0.38 in the Danish [10] and 0.4 and 0.36 in the British, respectively [6].

Interestingly, all these data showed that allelic distribution in our sample is closer to Saudi and Mauritanian populations than to the East-Central Tunisians. These findings reveal the genetic heterogeneity of the Tunisian population which is made of a mosaic of communities from different ethnic background (Arab, Berber, Roman, etc.). This population stratification was additionally maintained by a high prevalence of endogamous unions [24]. The observed MAF similarities between the Tunisian and Saudi might be explained by the Arab conquest in Tunisia that started in the 7th century, followed by a massive Bedouin invasion during the 11th century [25] that has largely contributed to the Tunisian gene pool. In the same context, the Mauritanian study performed by Abdelhamid et al. [22] explained the similarity of their K minor allele frequencies with the Saudi by the genetic affinities between the Moor group (80% of Mauritanians) and Arabs [22, 26].

On the other hand, findings from Mtiraoui et al. [20] showed that East-Central Tunisians are most related to Caucasians. These genetic similarities probably correspond to the well-known historical European influence in this region [27, 28], thus explaining the variability of the genetic background between the coastal regions of central Tunisia and the rest of the country. In this context, one study on the mitochondrial DNA diversity of the population of Monastir showed the relative predominance of Eurasian haplogroups (63%) in the East-Central areas of Tunisia (unpublished data).

When performing a “Tunisian” meta-analysis (Figure 1), the E23K variant was still associated with a 1.29-fold increased risk of T2DM (*P* < 10^−3^). We have also performed allelic meta-analysis in Arab population given the fact that several meta-analyses have reported the association of E23K with an increased T2DM risk in Caucasians and Asians [6, 9–11, 13, 29]. We found that the E23K variant is strongly associated with T2DM. Our results are consistent with previous meta-analyses among different ethnic groups where the E23K variant is considered to be a strong candidate variant for T2DM worldwide. In this context, two Caucasian meta-analyses [10, 29], grouping 4 studies (2824 subjects) and 18 studies (7768 subjects), respectively, reported similar results, with a significant association with T2DM, under a recessive model (OR = 1.49, *P* = 0.00022) and additive model (OR = 1.44, *P* = 0.0007), respectively. Likewise, two East Asian meta-analyses conducted by Zhou et al. [13] and by Takeuchi et al. [30] grouping, respectively, 8 studies (15503 subjects) and 7 studies (8217 subjects) showed association with an OR of 1.15 (*P* = 3 × 10^−9^) and an OR of 1.168 (*P* < 10^−3^), respectively. More recently, a global meta-analysis based on 49 case-control studies [31] reported a significant association with T2DM under a random-effects model, with per-allele odds ratio of 1.13 (*P* < 10^−5^). Significant results were found in only East Asians and Caucasians after ethnic stratification. Unfortunately, in the former study, the association in Arab populations has not been tested according to their ethnicity.

In conclusion, our case-control study confirmed that the* KCNJ11* E23K variant is significantly associated with the susceptibility to T2DM in the Tunisian population. Further genetic and functional studies are required to identify the relevant variant(s) at this locus and to investigate their effect(s) on T2DM susceptibility. To our knowledge, this study represents the first meta-analysis of E23K variant in* KCNJ11* gene in association with T2DM in Arab populations and complement previous meta-analyses in Asians and Caucasians. Nevertheless, due to our small sample size, further larger investigations and meta-analysis are needed to confirm the role of the E23K variant in genetic predisposition to T2DM in Arab populations.

## Figures and Tables

**Table 1:** Clinical and biochemical characteristics of study subjects.

Characteristics	T2DM (*n* = 250)	Control (*n* = 267)	*P* value
Gender (men/women)	94/156	105/162	0.65∗
Age (years)	60.2 ± 10.5	53.7 ± 11.6	<10^−4^^†^
Duration of diabetes (years)	14.31 ± 9.26	—	—
Age at diagnosis (years)	45.6 ± 9.5	—	—
HbA_1c_ (% (mmol/mol))	9.1 ± 1.9 (76 ± 20)	—	—
Height (cm)	161.9 ± 9.7	162.24 ± 9.6	0.71^†^
Weight (kg)	75.4 ± 13.7	74 ± 13.6	0.3^†^
Mean BMI (kg/m^2^)	28.9 ± 5.5	28.2 ± 5.3	0.2^†^
Systolic blood pressure (mmHg)	14.5 ± 2	13.4 ± 2.2	<10^−4^^†^
Diastolic blood pressure (mmHg)	8.3 ± 1.1	8.1 ± 5.2	0.75^‡^
Fasting plasma glucose (mmol/L)	11.5 ± 4.1	5.2 ± 0.5	<10^−4^^‡^
Total cholesterol (mmol/L)	5.0 ± 1.1	5.0 ± 1	0.95^†^
Triglycerides (mmol/L)	1.7 ± 0.9	1.3 ± 0.6	<10^−4^^‡^
HDL-cholesterol (mmol/L)	1.2 ± 0.4	1.5 ± 0.4	<10^−4^^†^
LDL-cholesterol (mmol/L)	2.8 ± 0.9	3 ± 0.9	0.27^†^

Data are expressed as means ± SD (standard deviation). ∗Pearson's chi-square test. ^†^Student's *t*-test for equal variances. ^‡^Welch's *t*-test for unequal variances.

**Table 2:** Genotypic and allelic distribution of the E23K variant in Tunisian patients with Type 2 diabetes and control subjects.

Genotype distribution			Allele		Allelic exact test	Global model		Additive model		Dominant model		Recessive model	
	T2DM patients (94 men/156 women)	Control subjects (105 men/162 women)	OR (95% CI)	*P*		OR (95% CI)	*P*	OR (95% CI)	*P*	OR (95% CI)	*P*	OR (95% CI)	*P*
EE	142 (55/87) (56.8%)	173 (65/108) (64.79%)		0.063∗				1.00					
EK	89 (33/56) (35.6%)	86 (34/52) (32.21%)		0.416∗				1.44 (0.94–2.2)	0.092				
KK	19 (6/13) (7.6%)	8 (6/2) (3%)		0.019∗		1.6 (1.14–2.27)	0.007	3.62 (1.26–10.41)	0.017	1.6 (1.07–2.41)	0.023	3.18 (1.11–9.07)	0.031
MAF (K)	0.254	0.19	1.44 (1.07–1.93)	0.015^†^	0.017								
*P* value for HWE	0.32	0.69											

Genotype distributions are shown as number (%). MAF: minor allele frequency. *P values comparing genotype distribution between patients with Type 2 diabetes and control subjects. ^†^Allele-specific *P* values. Odds ratio (OR), 95% CI, and *P* values were from logistic regression analyses with additive, dominant, and recessive models controlling age, sex, and BMI as covariates. In additive models, ORs are expressed per difference in number of rare alleles.

**Table 3:** Effects of *KCNJ11* E23K variant on diabetes-related phenotypes.

Phenotype	T2DM group			ANOVA∗	Control group			ANOVA∗	T2DM and control subjects			ANOVA∗
	EE	EK	KK	*P* value	EE	EK	KK	*P* value	EE	EK	KK	*P* value
Fasting plasma glucose (mmol/L)	11.03 ± 3.89	12.49 ± 4.13	10.81 ± 4.48	**0.04**	5.26 ± 0.5	5.12 ± 0.5	5.5 ± 0.42	0.13	7.93 ± 3.93	9.32 ± 4.81	9.54 ± 4.53	0.03^†^
Mean BMI (kg/m^2^)	29 ± 5.48	28.84 ± 5.82	28.55 ± 4.48	0.94	28.24 ± 5.61	28.33 ± 4.5	27.17 ± 4	0.89	28.61 ± 5.55	28.63 ± 5.29	28.24 ± 4.32	0.95
Total cholesterol (mmol/L)	5.06 ± 1.07	5.02 ± 1.04	5.17 ± 1.25	0.88	5.01 ± 0.97	5.2 ± 0.96	4.7 ± 0.64	0.39	5.03 ± 1.01	5.09 ± 1	5.06 ± 1.15	0.89
Triglycerides (mmol/L)	1.75 ± 0.88	1.67 ± 0.81	2.03 ± 1.34	0.85^†^	1.33 ± 0.63	1.33 ± 0.5	1.22 ± 0.31	0.7^†^	1.52 ± 0.79	1.53 ± 0.71	1.85 ± 1.23	0.69^†^
HDL-cholesterol (mmol/L)	1.23 ± 0.42	1.21 ± 0.44	1.27 ± 0.29	0.95	1.49 ± 0.4	1.39 ± 0.4	1.26 ± 0.4	0.23	1.42 ± 0.42	1.33 ± 0.42	1.27 ± 0.32	0.26
Systolic blood pressure (mmHg)	14.49 ± 2.13	14.45 ± 1.97	14.37 ± 2.02	0.98	13.34 ± 2.14	13.47 ± 2.45	15 ± 1.41	0.34	13.92 ± 2.21	14.06 ± 2.22	14.5 ± 1.88	0.52
Diastolic blood pressure (mmHg)	8.39 ± 1.15	8.12 ± 1.14	8.09 ± 0.80	0.25	8.33 ± 6.3	7.7 ± 1.32	8 ± 0.82	0.53^†^	8.36 ± 4.47	7.95 ± 1.23	8.07 ± 0.78	0.32^†^
LDL-cholesterol (mmol/L)	3.01 ± 1.05	2.5 ± 0.61	2.65 ± 0.39	0.10^†^	2.90 ± 0.91	3.18 ± 0.87	2.94 ± 0.64	0.20	2.93 ± 0.95	2.97 ± 0.84	2.78 ± 0.50	0.83

Values are mean ± standard deviation. ∗ANOVA was used to compare geometric mean levels of continuous characteristics across genotypes. ^†^ *P* value of Kruskal-Wallis test for unequal variance.

**Table 4:** Results of multiple regression analysis under a dominant model.

Dependent variable	Independent variables	Stand beta∗	*t* ^†^	*P* value^‡^
Fasting plasma glucose (mmol/L)	*KCNJ11* E23K genotype	0.65	2.17	0.031
	Gender	0.03	0.09	0.926
	Age	−0.007	−0.55	0.585
	BMI	0.003	0.12	0.907
	T2DM (presence or absence)	6.43	21.65	*P* < 10^−3^

*Stand beta standardized beta-coefficient; ^†^ *t*-statistic; ^‡^ *P* value for the *t*-statistic.
